# Effects of a Self-Management Telehealth Program on Improving Strength and Hand Function in Systemic Sclerosis Patients: A Randomized Controlled Trial

**DOI:** 10.3390/life15071087

**Published:** 2025-07-10

**Authors:** Orathai Wantha, Ajanee Mahakkanukrauh, Siraphop Suwannaroj, Kwankaew Tuydaung, Nonglak Methakanjanasak, Kannika Srichomphu, Jinnaphat Kraipoj, Chingching Foocharoen

**Affiliations:** 1 Division of Nursing, Faculty of Medicine, Khon Kaen University, Khon Kaen 40002, Thailand; orawan1@kku.ac.th (O.W.); kwantu@kku.ac.th (K.T.); 2 Department of Medicine, Faculty of Medicine, Khon Kaen University, Khon Kaen 40002, Thailand; ajamah@kku.ac.th (A.M.); siraphop@kku.ac.th (S.S.); 3 Faculty of Nursing, Khon Kaen University, Khon Kaen 40002, Thailand; nonchu@kku.ac.th; 4 Department of Rehabilitation, Faculty of Medicine, Khon Kaen University, Khon Kaen 40002, Thailand; kannika@kku.ac.th (K.S.); jinnapat@kku.ac.th (J.K.)

**Keywords:** systemic sclerosis, scleroderma and related disorders, exercise, hand function, hand deformity, disability, clinical trials

## Abstract

Objective: A self-management telehealth program to improve hand strength and function in systemic sclerosis (SSc) patients may improve their quality of life, so we investigated its efficacy. Methods: A 6-week prospective randomized controlled study was conducted in adults with SSc with a hand mobility in scleroderma (HAMIS) score > 1 or a limited range of motion in at least one hand joint. Participants were randomly allocated into three groups for six weeks of health education: (a) typical education, (b) watching video guides as needed, in addition to typical education, and (c) watching video guides and receiving weekly telephone notifications, in addition to typical education. The endpoints were the differences in self-management behavior, HAMIS scores, hand grip strength (HGS), and quality of life (QoL) using the European Quality of Life-5 Dimensions between groups, as well as the changes in these parameters compared to baseline. Results: A total of 24 patients per group were enrolled, with the majority diagnosed with diffuse cutaneous SSc (79.2%). Six weeks post-intervention, HGS improved significantly in both the video guide and telephone notification groups compared to typical education in both hands (*p* = 0.028, *p* = 0.044). Pincer grip differed between the groups in the non-dominant hand. Household modifications were more frequent in the video guide and telephone notification groups than in the typical education group (*p* = 0.023). All groups showed significant improvements in HGS and HAMIS scores in both hands, as well as in self-management behaviors, compared to baseline. QoL, as measured using a visual analog scale, improved significantly after the intervention in both the video guide and telephone notification groups, but not in the typical education group. Conclusions: Self-management telehealth programs effectively enhance hand strength, function, and self-management behaviors in patients with SSc with limited hand function. Weekly telephone notifications further reinforced continuity and engagement in these patients.

## 1. Introduction

Systemic sclerosis (SSc) is an uncommon autoimmune disease that affects connective tissue, marked by an overproduction of collagen by fibroblasts in the skin and internal organs, frequently involving the hands [1]. Hand dysfunction in SSc arises from a variety of factors, including inflammatory arthritis, joint contractures, tendon friction rubs, Raynaud’s phenomenon (RP), digital ulcers (DU), swelling of the hands, skin thickening, acro-osteolysis, and the development of calcinosis [1,2]. Contractures and deformities in the hand are the most common musculoskeletal impairments [1,3]. These manifestations, which often coexist, can contribute to difficulty with daily living and work activities [4], eroding quality of life (QoL) [2,5,6].

Current strategies to maintain and improve SSc hand function include paraffin wax treatment and hand stretching [3,7,8,9,10,11]. Most of the programs include face-to-face sessions with healthcare providers or caregivers; this is a limitation for patients who live far from the hospital and have economic problems [12]; however, the majority of studies involved small sample sizes and no control groups [13,14].

Self-management and telehealth programs seem to increase adherence in other chronic diseases [15,16]; however, few studies have been conducted on patients with SSc. Enhancing patient educational materials, such as concise video content featuring animations and SSc patients, makes them more enjoyable for patients. This study aimed to explore a telehealth-based self-management program, comprising self-monitoring, performing specific tasks to control disease and prevent complications, information-seeking, and self-adjustment based on disease activity, compared to typical education with and without video guidance for improving hand strength and function in patients with SSc.

## 2. Methods

This prospective randomized controlled study was conducted on adult patients with SSc who fulfilled the American College of Rheumatology/European Alliance of Associations for Rheumatology 2013 classification criteria for SSc [17] and attended the Scleroderma Clinic, Khon Kaen University, between October 2023 and August 2024. All patients were able to communicate in the Thai language, had stable disease, had hand involvement defined as a hand mobility in scleroderma (HAMIS) score ≥ 1 or a limited range of motion in at least one hand joint, had a smartphone capable of recording video clips, and were able to self-access videos on their smartphone. We excluded patients with overlap syndromes involving any other connective tissue diseases or who had severe contracture deformity of the hands (defined as a HAMIS score of 27), impaired hand sensation, cognitive impairment, hearing or visual impairment, physical limitations (e.g., paralysis, muscle weakness), surgical schedules related to the hand, eyes, or ears, required hospitalization within 6 weeks, or had infected wounds or active inflammation on the hand, wrist, or fingers that made them unable to perform hand exercises.

All participants meeting the inclusion criteria were randomly allocated by computer-generated random numbers into three groups by a block of 6 in a 1:1:1 ratio for 6 weeks of the self-management program: (a) Group A (typical education by a personalized supervised self-management program), (b) Group B (video guide as needed + typical education), (c) Group C (video guide + weekly telephone notifications + typical education). The study endpoint was assessed 6 weeks after the program.

### 2.1. Intervention

Typical education included a self-management booklet based on the self-management framework [18] comprising four categories: (a) self-monitoring, which consisted of self-observation and self-recording of DU and RP; (b) performing special tasks to manage the disease, including keeping warm, maintaining moisture, and taking traumatic wound precautions; (c) information seeking; and (d) self-adjustment based on disease activity and treatment. The content was validated by 6 experts: two rheumatologists, 2 physical therapists, 1 rheumatology nurse, and 1 nursing professor specializing in self-management.

The video guide focused on hand and joint exercises that included a 3.27-min educational video on 6 basic daily hand exercises, including: (a) stretching wrist flexion and extension; (b) range of motion (ROM) ulnar and radial deviation; (c) ROM finger abduction and adduction; (d) finger pincer; (e) finger flexion and extension; and (f) finger grab. The video content was validated by the same six experts listed above. A physical therapist coached the patients on a home program for self-hand and joint exercises, along with a video guide. Patients could review the exercises as needed by watching the videos at any time. For their daily routine, a nursing coach instructed all participants to apply superficial heat by soaking their hands in warm water for approximately 15–20 min [19]. They then applied baby oil and performed hand exercises following the video guide. The exercises were performed carefully if aches occurred due to inflammation or skin ulcers. Patients were instructed to stretch beyond the feeling of tightness and hold the stretch for approximately 5–10 s. They repeated this process 5–10 times on each side, for a total time of approximately 20–30 min, once a day, at least 5 days a week, for 6 weeks.

Telephone notifications consisted of weekly calls to ensure continuity and engagement with hand exercises. Each call lasted approximately 10 min, providing problem-solving support, knowledge reinforcement, and motivation to encourage adherence to the program.

Participants randomly assigned to Group A (the typical education group) received only standard educational care. Those randomly assigned to Group B (the video guide group) received both typical education and access to instructional videos to watch as needed. Participants randomly assigned to Group C (the video guide plus weekly telephone notification group) received typical education, access to instructional videos, and weekly notifications. Allocation concealment was achieved by using sequentially numbered, opaque, sealed envelopes containing group assignments. Investigators enrolling participants were unaware of the allocation sequence and opened an envelope only after each participant was enrolled, ensuring that the intervention group assignment (Group A, B, or C) was concealed prior to randomization and reducing the risk of selection bias.

### 2.2. Data Collection

Baseline assessment included medical history, demographic data, clinical characteristics of SSc, self-management using the Self-Management Behavior Questionnaire, QoL using the European Quality of Life-5 Dimensions (EQ-5D), hand function using the HAMIS, and hand grip strength test.

The Self-Management Behavior Questionnaire comprised an 11-item questionnaire using a 4-point Likert scale (1 = never, 4 = always) to assess patients based on the self-management framework [18]. The total score ranges from 11 to 44. Higher scores indicate better self-management behavior. The questionnaire was approved by 5 experts, with a content validity index of 0.80, tested in a pilot study with 10 patients with SSc, and demonstrated Cronbach’s alpha reliability of 0.78. The Thai version of the EQ-5D incorporates patient-reported outcomes across the domains of mobility, self-care, typical activity, pain or discomfort, and anxiety or depression, as well as a health status self-assessment via a visual analog scale (VAS) ranging from 0 to 100. The HAMIS includes 9 items that assess finger flexion and extension, thumb abduction, pincer grip, finger abduction, volar flexion of the wrist, dorsal extension, and pronation and supination of the forearm. Each item is scored from 0 (fully performed) to 3 (unable to perform) [20]. The total score ranges from 0 to 27, with higher scores indicating worse hand mobility. Internal consistency, measured by Cronbach’s alpha from a pilot study with 10 patients with SSc, was 0.80 to 0.85. Hand grip strength was measured using a goniometer (Camry digital hand dynamometer—90 kg or 200 lbs) in both hands and recorded for the dominant and non-dominant hand. The procedure was conducted in a sitting position with relaxed arms. Three tests were performed for each hand, and the highest value was recorded.

The primary endpoint was a comparison of hand function using the HAMIS score, self-management behavior, QoL, and hand grip strength between the groups 6 weeks after the intervention. The secondary endpoints included changes in hand function, self-management behavior, quality of life (QoL), and handgrip strength at 6 weeks post-intervention compared to baseline values. Patients were withdrawn if they had active inflammation or infected wounds on the hand, wrist, or fingers that affected their ability to perform the hand exercises.

The outcome assessments were performed by an assessor who was blinded to participants’ group assignments. The assessor was not involved in the randomization or intervention processes and was unaware of which intervention each participant received.

### 2.3. Operational Definitions

SSc was classified as the limited cutaneous SSc (lcSSc) or diffuse cutaneous SSc (dcSSc) subset, as per LeRoy et al. [21]. The onset of the disease was the date of the first non-Raynaud’s symptoms. RP referred to episodic digital ischemia provoked by cold or emotion, classically described as triphasic (pallor followed by cyanosis and then hyperemia), accompanied by numbness and pain [22]. DU is a painful denuded area with well-demarcated borders located on the volar aspect of the fingers. Hand deformity manifests as contractures of the finger joints, creating a claw-like appearance. Hand dysfunction was characterized by challenges performing work-related tasks or everyday activities [2]. Interstitial lung disease (ILD) is identified by the presence of interstitial scarring detected via high-resolution computed tomography [23]. Stable disease was defined as the absence of active disease or internal organ involvement that required urgent immunosuppressive treatment, dosage adjustments, or hospitalization.

### 2.4. Statistical Analyses

Baseline characteristics were summarized using descriptive statistics (percentages, means, standard deviations (SD), medians, and interquartile ranges (IQR)). Differences in continuous outcome variables between groups were assessed using one-way analysis of variance (ANOVA) or the Kruskal–Wallis test, as appropriate. Within-group differences were analyzed using paired *t*-tests or Wilcoxon signed-rank tests for continuous data and chi-square tests or Fisher’s exact tests for categorical data, as appropriate. All *p*-values were two-tailed, and statistical significance was set at *p*-value < 0.05. All statistical analyses were performed using STATA version 16.0 (Stata Corp, College Station, TX, USA).

### 2.5. Sample Size Calculations

The sample size was calculated based on recent literature, where the median HAMIS scores for the treatment and control groups were 2.0 (SD 1.0) and 3.0 (SD 1.0), respectively [12]. To detect a difference at 80% power and a significance level of 0.05, the sample size for each group should be 17 (overall 51). To compensate for the 30% dropout rate, we recruited 72 patients (24 patients per group) for this study.

## 3. Results

**Figure 1:**
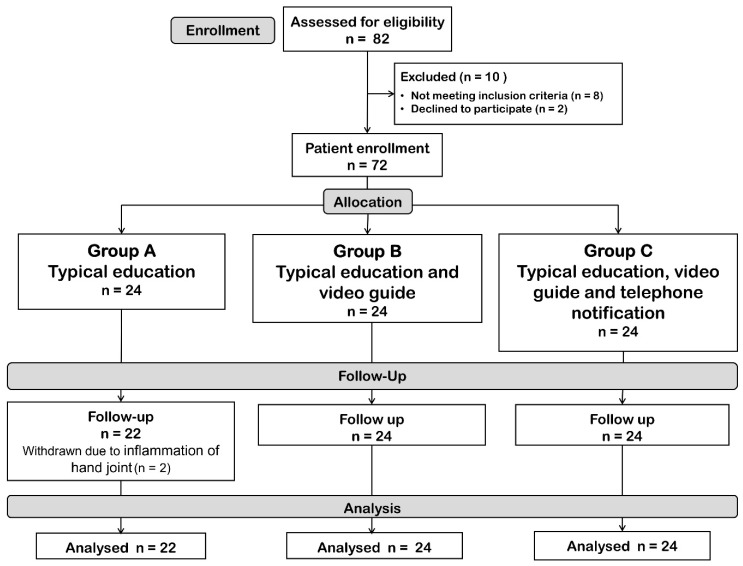
Study flow.

Eighty-two eligible patients were initially identified, but 10 were excluded because they did not meet the inclusion criteria (8 cases) and refused to participate in the study (2 cases). The remaining 72 patients were included in the study, with 24 patients randomly assigned to each group. Two patients withdrew from Group A (typical education), leaving 22 patients. The CONSORT diagram of the patient flow is presented in Figure 1.

Of a total of 72 SSc patients, the majority were the dcSSc subset (79.2%), with a female-to-male ratio of 2.6:1. The respective mean age and median disease duration were 55.9 ± 9.9 years and 5.9 years (IQR 2–9). Most participants (48, 66.7%) were employees. ILD was the most common internal organ involvement (44 cases, 68.6%), followed by gastrointestinal involvement (23 cases, 31.9%). The demographic and baseline characteristics of the overall sample and individual groups are presented in Table 1.

### 3.1. Primary Endpoint

Six weeks after the intervention, compliance with the video modules in Group B and engagement with telephone reminders in Group C were both above 80%. Hand grip strength was significantly improved in Groups B and C compared to Group A in both dominant and non-dominant hands (*p* = 0.028 and 0.044, respectively). The pincer grip also differed between the groups in the non-dominant hand. For hand care behaviors, the proportion of patients who made household modifications was higher in Group B than in other groups (*p* = 0.023). There were no statistical differences in HAMIS score and QoL between groups (Table 2).

### 3.2. Secondary Endpoints

Within-group comparisons of changes in the interested endpoints before and after the intervention revealed significant improvements in hand grip strength and HAMIS scores in both the dominant and non-dominant hands, as well as self-management behavior scores in all three groups. The EQ-5D-VAS score significantly improved in Groups B and C, but not in Group A. The details of the secondary endpoints are presented in Table 3.

## 4. Discussion

SSc is a chronic disease for which there is no definitive treatment to cure the disease [24]. Patients suffer from limitations in physical mobility and transformed hands, and nearly all daily activities are impaired by the disease [25]. Effective self-management involves making significant lifestyle modifications and behavioral changes, enabling individuals with SSc to live in a state of well-being despite their disease [26]. This study offers valuable insights into the effectiveness of telehealth-based self-management interventions in enhancing hand function among patients with SSc. Our findings demonstrate that incorporating technology-enhanced components into traditional educational approaches yields significant benefits for patients with hand involvement.

The primary aim of our study was to evaluate a comprehensive telehealth-based self-management program compared to typical education, with and without video guidance, for improving hand strength and function in patients with SSc. Our results showed that after 6 weeks of intervention, both Groups B and C (the video guide and video guide plus telephone notification groups) demonstrated significantly improved hand grip strength compared to the typical education group in both dominant and non-dominant hands.

Self-hand exercises, when used as a home program with the addition of video guides or telephone notifications, also improved hand function and grip strength compared with baseline. A home exercise program offers benefits not only in terms of saving time but also in reducing costs. Additionally, enhancing the program with self-learning tools, such as video guides and/or telephone notifications, can help and encourage patients to continue engaging with and performing hand exercises regularly. The incorporation of video guides in our intervention provided visual demonstrations that patients could review repeatedly. Although telehealth provides regular reinforcement, this approach is supported by research in other rehabilitation contexts, suggesting that video-based instruction enhances the learning of physical exercises. Previous studies have also demonstrated the effectiveness of home-based facial rehabilitation programs in patients with SSc. Maddali-Bongi et al. demonstrated that a combination of connective tissue massage and joint manipulation as a home exercise program improved facial skin thickness, increased mouth opening, and enhanced self-reported face- and mouth-related symptoms and function in SSc patients over a 9-week intervention period [27]. Bongi et al. conducted a randomized controlled trial that demonstrated superior outcomes from a comprehensive therapy combining joint manipulation of the wrist and fingers with a home program of active hand, wrist, and forearm range of motion (ROM) exercises compared to independent home exercises alone [28]. In addition, Karaaslan et al. conducted a randomized controlled study to compare the effects of real-time telerehabilitation and asynchronous telerehabilitation in SSc patients during the emergence of COVID-19 [29]. The finger ROM and hand function significantly improved in both groups that received real-time and asynchronous telerehabilitation after 8 weeks of interventions compared to the control group (individuals on the waiting list who received exercises only when specifically needed) [29]. The findings support the effectiveness of technology-based hand rehabilitation among patients with SSc. Our results suggest that telehealth components enhance the benefits of home exercise by providing regular reinforcement and visual demonstrations. This builds upon previous findings by demonstrating that comparable improvements can be achieved through a self-directed program, with the telehealth approach offering additional benefits for patients with mobility limitations or those living in remote areas. This finding is particularly significant because hand strength is a critical determinant of functional capacity in daily activities for patients with SSc.

The improvement in pincer grip function, particularly in the non-dominant hand, further emphasizes the effectiveness of the telehealth approach. A pincer grip is essential for fine motor tasks, such as buttoning clothes, writing, and handling small objects—activities that are commonly impaired in SSc patients with hand involvement [30]. The significant difference observed in this parameter suggests that the visual demonstration provided through video guides may enhance patients’ understanding and execution of exercises targeting specific hand movements.

The significant improvements in hand grip strength observed in our technology-enhanced intervention groups are consistent with findings by Piga et al., who reported improvements in hand function following a home-based rehabilitation program with periodic clinical supervision by telemonitoring [31]. Our study also demonstrated that these benefits can be achieved with remote supervision through video guides and telephone support, potentially reducing the need for in-person clinical visits.

Interestingly, while we observed significant improvements in hand grip strength and pincer grip, the between-group differences in the overall HAMIS scores did not reach statistical significance. This contrasts somewhat with Horvath et al., who found significant improvements in HAMIS scores following a comprehensive hand rehabilitation program [32]. This discrepancy may be due to the relatively short duration of our intervention (6 weeks) compared to longer programs (24 weeks) in the study, suggesting that continued intervention might yield further improvements in HAMIS scores over time.

Notably, patients in both technology-enhanced groups (Groups B and C: video guide and telephone notification plus video guide) were more likely to implement household modifications (83.3% and 91.7%, respectively) than those receiving typical education alone (50%, *p* = 0.023). This finding suggests that telehealth interventions may foster greater proactivity in disease self-management, encouraging patients to adapt their environments to accommodate their functional limitations.

While between-group comparisons revealed significant differences in hand grip strength and household modifications, all three groups demonstrated significant improvements from baseline in hand grip strength, HAMIS scores for both hands, and self-management behaviors. This suggests that even standard education has value, but the addition of technology-based support components enhances the outcomes.

QoL measurements, as assessed by EQ-5D-VAS, improved significantly from baseline in both the video guide and telephone notification groups, but not in the typical education group. The successful improvement in both functional outcomes and quality of life (QoL) suggests that telehealth approaches not only improved physical parameters but also translated to perceived improvements in overall well-being, a crucial outcome from the patient’s perspective.

Based on our investigation, the self-management program, whether delivered through typical education, video-guided instruction, or telemedicine support, showed benefits even within a short intervention period (6 weeks). However, we do not have information on the effect of disease duration and/or other clinical characteristics on treatment effectiveness, as the sample size was calculated based on the primary objective of the study and not for subgroup analyses. We are concerned that conducting subgroup analyses without a well-planned sample size calculation may result in low statistical power and, therefore, require caution when interpreting the results. In addition, compared to previous studies using intensive rehabilitation programs for patients with SSc [28,33], our telehealth approach potentially reduces healthcare resource utilization while maintaining clinical efficacy. However, we cannot conclude whether the intervention has long-term benefits. As disease progression or regression may occur during follow-up, and changes in medication could affect the results for patients requiring additional treatment due to disease progression, long-term outcomes in a randomized controlled trial may be influenced not only by our intervention, but also by other treatments, comorbidities, disease complications, and treatment compliance. The study by Rannou et al. revealed that a daily home exercise program, when continued for 1 month, failed to sustain benefits for disability at 12 months following the supervised, tailored program [33]. Additionally, 35% of participants experienced worsening of their disease. The authors proposed that non-sustained benefits might be related to poor compliance with the home exercise program. To evaluate the long-term sustainability of the intervention on hand function, we suggest conducting further research on telemedicine with at least 6 months of follow-up, in line with the existing literature. These patients may comply better with caregiver training, regular face-to-face sessions, and intermittent group treatment. Additionally, cost-effectiveness analyses comparing telehealth interventions with traditional care models should be conducted.

Advances in smartphone technology have been utilized to support patients in managing their chronic diseases [34]. Smartphone-based and web-based interventions appear to improve adherence among individuals with chronic conditions [15,35]. In this study, the “Hand and Joint Exercise Video” served as an instructional tool, combining animated visuals with text, and lasted only 3.2 min. Its concise format helps maintain patient attention throughout, leading to effective learning and a reported “good” level of satisfaction with the app. The video is available online, allowing open access beyond the study participants. However, we were unable to track how frequently or consistently the participants viewed the videos, which limited our ability to assess compliance with the self-management program. We recommend using a mobile application for future training programs, as it can log and record the date and time of user access, thereby improving compliance.

Although our study yielded promising results, several limitations should be considered. First, the 6-week intervention period was shorter than that of some previous studies. Short-term evaluations may not fully capture the extent of functional improvements or the durability of treatment effects, particularly for outcomes like hand function that might require a longer duration to show significant changes. Relying solely on short-term evaluation may underestimate or overestimate the true efficacy of an intervention. Future studies should consider including longer follow-up periods to more accurately assess the long-term impact and clinical significance of the intervention. Second, we excluded patients with severe contracture deformities and those without access to a smartphone or the Internet, which are necessary for utilizing video-based intervention. Consequently, we were unable to assess the effectiveness of video and telemedicine as an addition to the typical self-management programs for this subgroup. Third, our intervention primarily focused on exercise-based approaches and did not incorporate manual techniques, which have been efficacious in previous studies. By excluding manual techniques, our findings may not fully reflect the potential for maximizing functional gains in participants. A more holistic therapeutic approach that integrates both exercises and manual techniques should be considered in future studies. Fourth, participants could not be blinded to the type of intervention they received, due to the distinct nature of each intervention. This lack of participant blinding may introduce performance or expectation bias. Finally, this was a single-center study, which may limit the generalizability of the findings. These limitations highlight opportunities for future research that combines telehealth delivery with more comprehensive therapeutic approaches.

The strengths of our study include its randomized controlled design, comprehensive assessment of both objective (hand grip strength, HAMIS) and subjective (self-management behaviors and quality of life) outcomes, and the practical nature of the interventions that can be readily implemented in clinical practice. The incorporation of both video guides and telephone follow-ups provides insights into the relative benefits of different telehealth components.

These findings have important implications for clinical practice. Telehealth-based self-management programs offer a practical approach to enhancing hand function in patients with SSc, particularly in settings where in-person healthcare access is limited. The combination of educational materials, video guides, and telephone support provides a multimodal approach that addresses various aspects of patient education and engagement.

## 5. Conclusions

Our study demonstrates that telehealth-based self-management programs effectively enhance hand strength, function, and self-management behaviors in patients with SSc with limited hand function at 6 weeks after intervention. The addition of video guides improved outcomes compared to typical education alone, and weekly telephone notifications further reinforced continuity and engagement. These findings support the integration of telehealth components into standard care for patients with SSc to optimize hand function and quality of life. Further research should focus on longer follow-up periods, inclusion of patients with severe contracture deformities of the hands, and broader multicenter validation to ensure these interventions are accessible, effective, and sustainable in real-world practice.

## Figures and Tables

**Table 1:** Demographic and baseline characteristics of the overall sample and individual groups.

Clinical Characteristics		Total SSc n = 72	Intervention			*p*-Value *
			Group A Typical Education n = 24	Group B Video Guide n = 24	Group C Video and Telephone Notification n = 24	
Age (years), mean (SD)		55.9 (9.9)	56.1 (10.3)	53 (11.7)	58.8 (6.9)	0.14
Age at onset (years), mean (SD)		50.3 (11.2)	50.8 (11.6)	46.8 (12.8)	53.3 (8.3)	0.13
Disease duration (years), median (IQR)		5.9 (2–9)	6.1 (2–8.5)	6.2 (1.5–11)	5.6 (2–8.5)	0.92
Sex, n (%)						0.62
	Male	52 (72.2)	19 (79.2)	17 (70.8)	16 (66.7)	
	Female	20 (27.8)	5 (20.8)	7 (29.2)	8 (33.3)	
Education level, n (%)						0.95
	Primary-high school	36 (50)	13 (54.2)	11 (45.8)	12 (50)	
	Diploma	17 (23.6)	5 (20.8)	7 (29.2)	5 (20.8)	
	Bachelor’s or higher	19 (26.4)	6 (25)	6 (25)	7 (29.2)	
Occupation, n (%)						0.27
	Unemployed	24 (33.3)	11 (45.8)	6 (25)	7 (29.2)	
	Employee	48 (66.7)	13 (54.2)	18 (75)	17 (70.8)	
SSc subset, n (%)						0.78
	lcSSc	15 (20.8)	5 (20.8)	4 (16.7)	6 (25)	
	dcSSc	57 (79.2)	19 (79.2)	20 (83.3)	18 (75)	
Clinical presentation on study date, n (%)						
	Raynaud’s phenomenon	53 (73.6)	18 (75)	20 (83.3)	15 (62.5)	0.26
	Digital ulcer	24 (33.3)	9 (29.2)	7 (29.2)	10 (41.7)	0.57
	Salt and pepper skin	35 (48.6)	12 (50)	13 (54.2)	10 (41.7)	0.68
	Telangiectasia	21 (29.2)	9 (37.5)	6 (25)	6 (25)	0.55
Smoking, n (%)		4 (5.56)	0	3 (12.5)	1 (4.2)	0.68
Organ involvement, n (%)						
	Interstitial lung disease	44 (61.1)	13 (54.2)	16 (66.7)	15 (62.5)	0.67
	Gastrointestinal	23 (31.9)	6 (25)	9 (37.5)	8 (33.3)	0.64
	Heart	8 (11.1)	4 (16.7)	2 (8.3)	2 (8.3)	0.57
	Renal	2 (2.8)	1 (4.2)	0	1 (4.2)	0.59
	Arthralgia/Arthritis	1 (1.4)	1 (1.4)	0	0	0.36
	Muscle	9 (12.5)	5 (20.8)	1 (4.2)	3 (12.5)	0.22
Serology, n (%)						
	Anti-topoisomerase positive	60 of 67 (89.6)	20 of 22 (90.9)	20 of 22 (90.9)	20 of 23 (87.0)	0.88
	Anti-centromere positive	3 of 60 (5.0)	1 of 18 (5.6)	1 of 22 (4.6)	1 of 20 (5.0)	0.99
mRSS, mean (SD)		10.1 (8.5)	10.1 (8.6)	10 (6.5)	10.1 (10.5)	0.99
Hand grip strength (kg), mean (SD)						
	Dominant hand	18.3 (7.5)	15.8 (1.16)	19.2 (8.5)	19.9 (8.2)	0.16
	Non-dominant hand	17.2 (7.5)	15.5 (5.7)	17.5 (7.9)	18.9 (8.2)	0.19
HAMIS, mean (SD)						
	Dominant hand	6.3 (4.5)	6.6 (5.6)	6.1 (3.4)	6.3 (4.4)	0.91
	Non-dominant hand	4.9 (4.2)	5.2 (4.2)	5.0 (3.7)	4.6 (3.5)	0.87
QoL by EQ-5D, n (%)						0.34
	Mobility	20 (27.8)	7 (29.2)	9 (37.5)	4 (16.7)	0.55
	Self-care	27 (37.5)	11 (45.8)	10 (41.7)	6 (25)	0.29
	Daily activities	41 (56.9)	15 (62.5)	14 (58.3)	12 (50)	0.59
	Pain or discomfort	49 (68.1)	18 (75)	17 (70.8)	14 (58.3)	0.73
	Anxiety or depression	56 (77.8)	17 (70.8)	21 (87.5)	18 (75)	0.21
EQ-5D VAS, mean (SD)		67.9 (13.3)	71.6 (12.0)	64.4 (13.3)	67.7 (13.9)	0.16
Self-management behavior, mean (SD)		29.0 (5.4)	28.1 (4.5)	29.8 (6.3)	29.2 (5.4)	0.55

* Comparison between typical education, video guide-adding, and video-telephone notification adding group; SD: standard deviation; IQR: interquartile range; SSc: systemic sclerosis; lcSSc: limited cutaneous systemic sclerosis; dcSSc: diffuse cutaneous systemic sclerosis; mRSS: modified Rodnan skin score; HAMIS: hand mobility in scleroderma; QoL: quality of life; VAS: visual analog scale.

**Table 2:** Primary endpoints.

Clinical Characteristic			Intervention			*p*-Value *
			Group A Typical Education n = 22	Group B Video Guide n = 24	Group C Video and Telephone Notification n = 24	
Hand grip strength (kg); mean (SD)						
	Dominant hand		16.7 (5.4)	21.4 (9.1)	23.2 (9.1)	0.172 ^a^, 1.00 ^b^, 0.028 ^c^*
	Non-dominant		16.9 (5.4)	20.3 (8.4)	22.7 (9.0)	0.471 ^a^, 0.827 ^b^, 0.044 ^c^*
HAMIS, mean (SD)						
	Dominant hand		5.5 (5.9)	2.9 (3.0)	3.7 (4.5)	0.25
		Finger flexion	0.50 (0.74)	0.25 (0.53)	0.25 (0.68)	0.31
		Finger extension	0.73 (0.93)	0.63 (0.87)	0.63 (0.71)	0.67
		Thumb abduction	0.74 (0.91)	0.25 (0.44)	0.38 (0.77)	0.38
		Pincer grip	0.86 (0.88)	0.58 (0.77)	0.54 (0.78)	0.64
		Finger abduction	0.41 (0.73)	0.08 (0.28)	0.29 (0.55)	0.29
		Volar flexion	0.50 (0.74)	0.21 (0.51)	0.33 (0.56)	0.46
		Dorsal extension	0.64 (0.84)	0.33 (0.48)	0.17 (0.38)	0.15
		Pronation	0.68 (1.04)	0.17 (0.38)	0.50 (0.93)	0.21
		Supination	0.77 (0.92)	0.42 (0.65)	0.75 (0.79)	0.46
	Non-dominant		4.0 (5.1)	2.6 (3.3)	1.6 (2.4)	0.08
		Finger flexion	0.41 (0.66)	0.33 (0.64)	0.13 (0.34)	0.49
		Finger extension	0.50 (0.86)	0.42 (0.88)	0.33 (0.56)	0.46
		Thumb abduction	0.40 (0.84)	0.21 (0.41)	0.13 (0.34)	0.27
		Pincer grip	0.82 (0.85)	0.58 (0.78)	0.21 (0.65)	0.035 *
		Finger abduction	0.36 (0.73)	0.08 (0.28)	0.22 (0.51)	0.38
		Volar flexion	0.45 (0.67)	0.25 (0.53)	0.17 (0.38)	0.45
		Dorsal extension	0.55 (0.80)	0.25 (0.44)	0.13 (0.34)	0.27
		Pronation	0.27 (0.63)	0.13 (0.34)	0.04 (0.20)	0.23
		Supination	0.45 (0.59)	0.37 (0.49)	0.33 (0.56)	0.74
QoL by EQ5D, n (%)						
	Mobility		6 (27.3)	8 (33.3)	4 (16.7)	0.39
	Self-care		8 (36.4)	7 (29.2)	4 (16.7)	0.31
	Daily activities		9 (40.9)	6 (25)	6 (25)	0.40
	Pain or discomfort		14 (63.6)	13 (54.2)	9 (37.5)	0.43
	Anxiety/ depression		6 (27.3)	8 (33.3)	4 (16.7)	0.21
EQ5D VAS, mean (SD)			73.8 (11.5)	75.6 (12)	79.2 (12.7)	0.32
Hand care behaviors						
	Exercise habit, mean (SD)		3.5 (0.6)	3.8 (0.3)	3.9 (0.3)	0.15
	Keep warm, mean (SD)		2.5 (1.1)	3.1 (0.9)	3.1 (0.8)	0.19
	Skin moisture, mean (SD)		3.6 (0.6)	3.7 (0.8)	3.7 (0.6)	0.68
	Hand injury precautions, mean (SD)		3.8 (0.4)	4 (0)	4 (0.2)	0.014 *
	Household modifications, mean (SD)		3.5 (0.5)	3.5 (0.9)	3.8 (0.6)	0.023 *
	Emotional management, mean (SD)		3.6 (0.6)	3.9 (0.3)	3.9 (0.3)	0.11

a: comparison between typical education and video guide-adding group; b: comparison between video guide and telephone adding group; c: comparison between typical education and telephone adding group. * statistically significant (*p* < 0.05). SD: standard deviation; HAMIS: hand mobility in scleroderma; QoL: quality of life; VAS: visual analog scale.

**Table 3:** Secondary endpoints.

Clinical Characteristic		Group A Typical Education n = 22				Group B Video Guide n = 24				Group C Video and Telephone Notification n = 24			
		Baseline	Week 6	Mean Difference (95%CI)	*p*-Value	Baseline	Week 6	Mean Difference (95%CI)	*p*-Value	Baseline	Week 6	Mean Difference (95%CI)	*p*-Value
Hand grip strength (kg); mean (SD)													
	Dominant hand	15.8 (5.4)	16.7 (5.4)	−1.0 (−1.6, −0.3)	0.004 *	19.2 (8.5)	21.4 (9.2)	−2.2 (−3.1, −1.3)	<0.001 *	19.9 (8.2)	23.2 (9.1)	−3.3 (−4.3, −2.3)	<0.001 *
	Non-dominant	15.5 (5.7)	16.9 (5.4)	−1.4 (−2.2, −0.6)	0.001 *	17.5 (7.9)	20.3 (8.4)	−2.8 (−3.4, −2.1)	<0.001 *	18.9 (8.2)	22.7 (9.0)	−3.8 (−4.6, −2.9)	<0.001 *
HAMIS, mean ± SD													
	Dominant hand	6.7 (5.8)	5.6 (6.0)	1.1 (0.3, 1.9)	0.01 *	6.1 (3.4)	2.9 (3.0)	3.2 (2.3, 4.1)	<0.001 *	6.3 (4.4)	3.8 (4.5)	2.5 (1.7, 3.3)	<0.001 *
	Non-dominant	5.3 (5.6)	4.1 (5.1)	1.2 (0.4, 2.0)	0.01 *	5.0 (3.7)	2.6 (3.4)	2.4 (1.4, 3.4)	<0.001 *	4.6 (3.5)	1.7 (2.4)	3.0 (1.9, 4.0)	<0.001 *
QoL by EQ-5D		8 (1.8)	7.3 (1.6)	0.5 (−0.1, 1.2)	0.076	8.2 (1.8)	7.2 (1.5)	1.1 (0.6, 1.6)	<0.001 *	7.5 (1.4)	6.5 (1.5)	1.0 (0.5, 1.4)	<0.001 *
EQ-5D VAS, mean ± SD		72.7 (11.6)	73.9 (11.5)	−1.1 (−2.1, −0.2)	0.025 *	64.4 (13.3)	75.6 (12)	−11.3 (−13.5, −9.0)	<0.001 *	67.7 (13.9)	79.2 (12.7)	−11.5 (−13.0, −9.9)	<0.001 *
Self-management behavior, mean ± SD		28.1 (4.5)	38.2 (4.2)	−10.2 (−12.2, −8.2)	0.001 *	29.8 (6.3)	40.9 (3.9)	−11.1 (−13.7, −8.5)	0.001 *	29.2 (5.4)	42.9 (3.1)	−13.8 (−16.3, −11.2)	0.001 *

* statistically significant (*p* < 0.05). SD: standard deviation; HAMIS: hand mobility in scleroderma; QoL: quality of life; VAS: visual analog scale.

## Data Availability

The datasets used and/or analyzed in the current study are available from the corresponding author upon reasonable request.
